# The Influence of Hierarchical Masks on Masked Repetition Priming: Evidence From Event-Related Potential Investigation

**DOI:** 10.3389/fnhum.2019.00070

**Published:** 2019-03-06

**Authors:** Ying Mei, Yuqian Dai, Yi Lei

**Affiliations:** ^1^Research Center for Brain Function and Psychological Science, Shenzhen University, Shenzhen, China; ^2^Faculty of Education and Psychology, University of Jyvaskyla, Jyvaskyla, Finland; ^3^Shenzhen Key Laboratory of Emotion and Social Cognitive Science, Shenzhen University, Shenzhen, China; ^4^Center for Language and Brain, Shenzhen Institute of Neuroscience, Shenzhen, China

**Keywords:** conceptual hierarchical relationship, fluency, familiarity, recognition, P2, FN400

## Abstract

The discussion about relationship between prime and target has contributed to the mechanism of priming effect and object recognition. Nevertheless, the role of relationship between mask and target in those cognitive processes remains unquestioned. In the present study, we aim to investigate how mask-target hierarchical relationship may affect word priming and familiarity, by using the masked repetition paradigm and manipulating three hierarchical relationship between mask and target. It is hypothesized that a closer hierarchical relationship between mask and target is associated with a higher mask target similarity, and thereby it leads to a worse recognition performance. Our behavioral results do not support this hypothesis by showing no effect of mask target hierarchical relationship on response time (RT) and accuracy. Event-related potentials (ERPs) indicated that highly similar mask-target triggered (i.e., the subordinate-subordinate-subordinate trials) larger N1 amplitudes, suggesting that it requires more cognitive resource to discriminate the stimuli. In addition, trials with highly similar mask-target hierarchical relationship induced smaller P2 (150–250 ms) and larger mid-frontal FN400 amplitudes than do trials with low mask-target similarity (i.e., the subordinate-basic-subordinate and the subordinate-superordinate-subordinate trials). Our results suggested that the similarity between mask and target may impede conceptual fluency to reduce word priming and familiarity effect.

## Introduction

In the majority of investigations of word recognition processes, masked priming has become a key tool to study word priming and memory related familiarity. Numerous studies have shown that more accurate and faster behavioral responses to target words, when target words are preceded by semantically related prime (e.g., cat-dog) or by identity word (e.g., dog-dog) relative to when they are preceded by semantically unrelated prime (hand-dog). This priming and familiarity effect are considered to be products of semantic relationships or associative links between primes and targets (Neely, 1991; Dehaene et al., 1998; Kouider and Dehaene, 2007; Ortells et al., 2016). Specifically, these effects started by the masked prime and then modified by the similarity between subsequent target and prime. Although previous research has explored the semantic priming effects under different between prime-target relationships, but how do different semantic mask affect priming and familiarity has received little interest.

In a backward visual masking paradigm, the aftercoming mask impairs the further processing of the earlier prime, and then the mask stimulus itself masked by the target. The masking effect could be interpreted as the mask and target are “fused” and are treated as one stimulus, which result in recognition impairment (Turvey, 1973). The degree of similarity between the mask and target (share same feature) will largely affect the recognition. Hence, a pattern mask (e.g., a random letter string, symbols, or scrambled patterns) is most effective when its component overlapped with the masked stimulus (e.g., same length, angle, place). Naish (1980) demonstrated that this effect was not only existing in low feature-detecting level, but also semantic feature-detecting level. It is shown that when the mask is semantically related to the target, the processing to the target can be reduced. However, the discussion about the target and mask have made a little progress in recent decades. The majority works have been focused on exploring the relationship between prime and target.

Typically, although the masked prime word flashes too quickly to be perceived, it is still able to promote the processing of word recognition. This promotion effect is smaller than when target following supraliminal primes (Forster and Davis, 1984). The priming effect somewhat can reflect an ease fluency processing (Woollams et al., 2008). The dual-process model of recognition memory suggests that recognition requires remembering specific details of items (i.e., recollection) and recognizing that a given item has been presented previously (i.e., familiarity; Yonelinas, 1999, 2002). A substantial parallel literature on masked repetition priming (Jacoby and Whitehouse, 1989; Rajaram, 1993; Westerman, 2001, 2008; Westerman et al., 2002; Kurilla and Westerman, 2008) have found that the fluency affects recognition: highly perceptual or conceptual fluency triggered more old judgment (Rajaram, 1993; Rajaram and Geraci, 2000). By using masked repetition priming paradigm combined with remember/know (R/K) paradigm, some studies showed that processing fluency only work on familiarity, not recollection (Rajaram, 1993; Huber et al., 2008; Woollams et al., 2008; Bruett and Leynes, 2015). Put it together, it is believed that the increased perceptual and conceptual fluency processing has contributed to the familiarity and some kinds of priming (Whittlesea and Williams, 2000; Yonelinas, 2002; Woollams et al., 2008).

The present study aimed to investigate the electrophysiological correlates of how different concept level masks to affect processing fluency and subsequent recognition by recording event-related potential (ERP) responses. In order to control the priming effect caused by the different prime word and target word, the current study adopted a masked repetition priming paradigm. Typically, more accurate and faster response time (RT) would be observed in repeated as opposed to non-repeated condition (Jacoby and Whitehouse, 1989; Misra and Holcomb, 2003). As mentioned above, this paradigm has made a great contribution to processing fluency in recognition memory. Briefly, previous study varied the perceptual features (e.g., font and size, Chauncey et al., 2008; clarity, Andrew Leynes and Zish, 2012; typography, Jacoby and Hayman, 1987) or semantic features (e.g., conceptual meaningfulness, Li et al., 2015; predictability of a sentence, Whittlesea and Williams, 2000; word frequency, Rajaram and Neely, 1992) to manipulate the fluency level. However, how do vary mask affect fluency and recognition attracts little attention. Typically, unmasked repetition priming (more-fluently processed) led to attenuation of recognition memory than masked repetition (Misra and Holcomb, 2003). How different level of mask affect fluency and recognition is still a blank to our knowledge. Hence, the present study manipulated the conceptual hierarchical relationship between the mask and target to see whether variate fluency and recognition effect were to be elicited. The advantage of employing conceptual hierarchical relationship is that members from one category can avoid deviations caused by different conceptual properties. For example, Kiefer (2005) found artifactual (e.g., tools) and natural categories (e.g., animals) interacted with the ERP repetition effect.

Conceptual knowledge can be categorized hierarchically according to abstractness (Rosch, 1988). A basic-level category (e.g., “bird”) is more specific than its superordinate-level category (e.g., “animal”) but more abstract than its subordinate categories (e.g., “sparrow”; Clarke and Tyler, 2015). The semantic similarity between concepts determines their relatedness (Markman and Wisniewski, 1997; Resnik, 1999). It is assumed that the more specific the concepts are, the more similar to each other they are (Markman and Wisniewski, 1997). Therefore, subordinate-level concepts are more similar to each other than basic-level concepts, and basic-level concepts are more similar to each other than superordinate-level concepts. For example, most birds (e.g., sparrows and ravens) share features like having wings and beaks and laying eggs, although different birds have different details in these features. However, mammals share few of these features (Murphy and Brownell, 1985; Morris and Murphy, 1990; Markman and Wisniewski, 1997). Hence, the two subordinate concepts sparrow and raven are more similar to each other than the two basic-level concepts mammal and bird. According to the structure of conceptual hierarchical knowledge, three level of similarity between mask and target can be manipulated: highly similar (e.g., subordinate-subordinate), medium similar (e.g., basic-subordinate), low similar (e.g., superordinate-subordinate).

ERP studies investigating category-related brain activations and masked repetitions priming effects have provided some insight into the electrophysiological correlates of concept-related priming, fluency and familiarity (Kiefer, 2005; Hoenig et al., 2008; Wang et al., 2015; Bader and Mecklinger, 2017). Three ERP component may be involved in those processes: N1, P2 and FN400. The fronto-central N1 amplitudes is sensitive to concept feature attribution processing (Hoenig et al., 2008; Lin and Chan, 2018). For example, Hoenig et al. (2008) tested the conceptual flexibility by manipulating concept features (visual, action-related) for two concept categories (artifactual and natural objects). The results showed a rapid modulation to concept features rather than later concept category processing: largest N1 peaks were found when action attributes prime for natural category target at fronto-central regions (116 ms) and when visual attributes prime for nature category target at occipito-parietal regions (150 ms), respectively. Followed study (Lin and Chan, 2018) also discovered similar concept feature regulation effect: the N1 (110–160 ms) was larger for targets primed by functional features than sensory feature both for nature and artificial category. This effect was even stronger at anterior sites than posterior region. In the present study, the concept hierarchical relationship between mask and target are based on concept shared features (Morris and Murphy, 1990). Hence, we hypothesize that the N1 amplitude would be modulated when targets are preceded by different levels of shared-feature masks.

Previous ERP studies have demonstrated that semantic priming and familiarity are functionally different processes and were indexed by central parietal N400 and mid-frontal FN400, respectively (Bridger et al., 2012). However, when priming and recognition tasks confounded together, the FN400 and N400 component share similar frontal distribution (Bridger et al., 2012; Stróżak et al., 2016; Leynes et al., 2017). The 300–500 ms FN400 component at mid-frontal electrodes is associated with an old/new ERP different (old ERP is more positive than new). In the semantic priming paradigm, the N400 priming effect is known as the phenomenon that N400 amplitude to target is less negative for semantic related/congruent prime-target combination compared with unrelated/incongruent pairings (Ortells et al., 2016).

In addition, frontal-distributed P2 component that co-occur with the N400 effect is thought to reflect perceptual fluency (Andrew Leynes and Zish, 2012; Li et al., 2015; Bader and Mecklinger, 2017). Usually, the lager positive old/new effect in P2 time window, the smaller negative old/new effect in N400 time window. Hence, the enhanced P2 and attenuated N400 indicates that fluency affects subsequent recognition by increasing fluency processing, which leads more familiarity responses. We hypothesize that the hierarchical relationship between mask and target would also modulate the priming and recognition memory represented by priming, fluency and familiarity related ERP component (P2, mid-frontal FN400).

In the current study, the prime is presented very briefly and is then quickly replaced by the mask, which lead to an unawareness of the priming. However, the presentation of the mask and target are supraliminal, which generate a processing the relationship between the mask and target. When the mask is highly semantically similar to the target, it becomes more difficult for the subject to distinguish them apart in a short time (Naish, 1980), and thus interfere with the processing of fluency and recognition. We hypothesized that the strength of the masks’ interference may depend on the similarity between the mask and the target stimuli. The interference can be reflected by the reduction of recognition accuracy and the increase in RT. We expect to observe more interference for high-similarity mask-target pairs than for low-similarity pairs.

## Materials and Methods

### Participants

Twenty-one right-handed healthy volunteers (11 females), between 18 and 26 years old (21.68 ± 1.96, mean ± SD), took part in the main experiment. All participants reported normal or corrected-to-normal vision and normal color perception. All participants gave written informed consent and were paid for their participation. The local ethics committee of Shenzhen University (Shenzhen, China) approved the procedure and the methods complied with the relevant guidelines and regulations. In addition, all participants were aware of the experimental purpose.

### Materials

The experimental materials were adapted from our previous studies (Lei et al., 2010, 2017). All words were presented in Chinese (Song Ti font). As shown in Table 1, we used “plant” and “animal” as the superordinate-level categories. There were two basic-level categories under each superordinate-level category (animal: birds and insects; plant: vegetables and fruits). Under each basic-level category, there were 10 subordinate-level categories. We controlled for word length, frequency and word typicality. All concepts consisted of no more than two Chinese characters. They were rated on typicality on a 5-point Likert scale (1 = not typical at all, 5 = very typical); only concepts rated as highly typical (*M* = 4.47, *SD* = 0.15) were included in the current study. Then the mean word frequency of the subordinate members was 72.73 times per 100,000 words based on the Corpus Word list^1^.

**Table 1:** Experimental materials.

Hierarchy of class concept	Materials
Superordinate level	animal, plant
Basic level	fruit, vegetable, bird, insect
Subordinate level	bird: swallow, magpie, sparrow, pigeon, oriole, kingfisher, tit, crow, wild geese, and lark
	insect: cockroach, ladybug, cricket, grasshopper, beetle, butterfly, bee, dragonfly, fly, and locust
	fruit: apple, orange, pear, peach, watermelon, banana, pineapple, tangerine, grape, and strawberry
	vegetable: cabbage, green vegetable, spinach, radish, cauliflower, eggplant, cucumber, lettuce, and celtuce

### Design

In the current study, we used within-subject conditions: the subordinate-subordinate-subordinate (sub-sub-sub) condition, the subordinate-basic-subordinate (sub-basic-sub) condition, the subordinate-superordinate-subordinate (sub-sup-sub) condition, and the control condition. In the three experimental conditions, we employed the repetition priming paradigm, where the prime and the target words were a same subordinate-level concept, and the mask word was a subordinate-, a basic-, or a superordinate-level concept that was semantically associated with the prime. The control condition is the same as the experimental condition, except for that the prime and the target were two different subordinate-level concepts. Each condition comprised 80 trials, and thus there were 320 experimental trials (presented randomly) in total. Prior to the eight experimental blocks, participants completed a training block of 40 trials to acclimatize to the task conditions.

### Procedure

**Figure 1:**
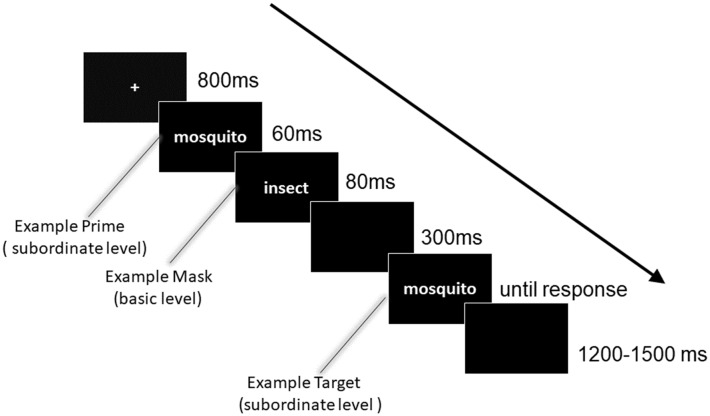
Experimental procedure of the masked priming paradigm. In the present design, four experimental conditions were included, i.e., subordinate- subordinate-subordinate, subordinate-basic-subordinate, subordinate-superordinate-subordinate, and control conditions. To avoid a fixed positive response tendency, priming and target stimuli were either identical or different. Participants were asked to judge whether the priming and target stimuli were identical or not by pressing “F” or “J” key on a standard QWERTY keyboard, or they could press the space bar if could not decide. The number of identical and different trials was the same. Note that the illustration depicts a subordinate masked by basic categorization. Moreover, “mosquito” was a typical representation of “insect.”

The stimuli were presented using E-Prime software (Psychology Software Tools, Inc. Pittsburgh, PA, USA) on a 17-inch computer monitor with a gray background. The viewing distance was approximately 60 cm. Responses were registered using a standard QWERTY keyboard. Specifically, to control for the fixed positive response tendency, the priming and target stimuli were either identical or different with equal probability. Participants were required to judge whether the priming and target stimuli were identical or different by pressing one of two keys (“F” or “J, ” using the left or right forefinger), or they could press the space bar if they could not decide. The assignment of the response keys was counterbalanced across participants. Participants were instructed to perform the task as quickly as possible without sacrificing accuracy. Figure 1 shows a representative sequence of one trial and the detailed timing of each stimulus. On each trial, the stimuli were presented as follows: (1) a fixation for 800 ms; (2) a priming stimulus for 60 ms; (3) a masking stimulus for 80 ms; (4) a blank interval for 300 ms; (5) a target stimulus until a key was pressed; and (6) a blank interval of 1,200–1,500 ms (the interval varied randomly).

### EEG Recording and Pre-processing

The EEG data were recorded using the 64-channel Brain Products system (Brain Products, Munich, Germany) according to the extended 10–20 system. The ground electrode was on the medial frontal line and the references were on the left and right mastoids. Horizontal electrooculograms (EOGs) were recorded from the orbital rims of both eyes. Vertical EOG was recorded from the above and below the left pupil. Data were acquired with a sampling rate of 500 Hz and online filtered with a band-pass of 0.01–100 Hz. Interelectrode impedance was below 5 kΩ.

The offline analysis of the EEG data was performed in MATLAB using EEGLAB and ERPLAB toolboxes (Delorme and Makeig, 2004; Lopez-Calderon and Luck, 2014). EEG data was filtered using IIR-Butterworth filters with half-power cutoffs at 0.1–30 Hz (roll-off = 12 dB/oct; Luck, 2014). Independent component analysis (ICA) was subsequently performed to correct components associated with eye movements and eye-blinks. The ICA-corrected EEG data were re-referenced to the average of the left and right mastoids (Luck, 2014). The control condition was excluded from epoch segmentation because it is incomparable with the other three conditions. EEG epochs were segmented and time-locked to the target stimulus in 1,000 ms time-windows (pre-stimulus 200 ms and post-stimulus 800 ms). Noisy trials were excluded using the moving window peak-to-peak amplitude method (Luck, 2014) with a window width of 200 ms, window step of 100 ms, and a 80-μV threshold.

### Data Analyses

Behavioral and ERP responses to the target stimuli were analyzed. To analyze the RTs and accuracy, one way repeated-measures analysis of variances (ANOVAs) were used. To analyze the mean amplitudes of P2 and FN400, two-factor repeated measures ANOVAs were used with condition (sub-sub-sub, sub-sup-sub, sub-bas-sub), and brain region (anterior, posterior) as within-subject factors. According to previous studies, mean amplitudes of FN400 were measured during a 300–400 ms time window after conclusion onset. The mean amplitudes of P2 were measured during a 150–250 ms time windows. In order to increase statistical strength and reduce false effects (Luck and Gaspelin, 2017), the F3, F1, Fz, F2, F4, FC3, FC1, FCz, FC2, and FC4 electrodes were collapsed by averaging their values as an indication of anterior activity; the CP3, CP1, CPz, CP2, CP4, P3, P1, Pz, P2, and P4 electrodes were also collapsed by averaging their values as an indication of posterior activity. For all analysis, the *p* values of *F*-test were corrected for deviations using the Greenhouse-Geissier method.

## Results

### Behavioral Performance

**Figure 2:**
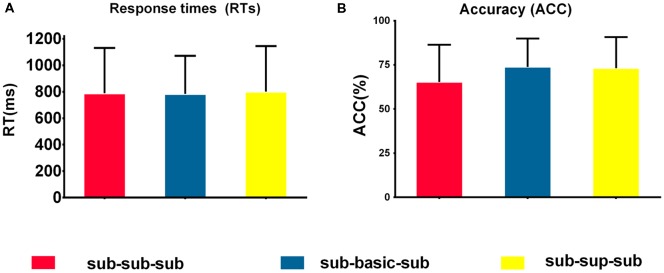
Behavioral performance. **(A)** Mean response times (RTs) to target stimuli in the three conditions. There were no significant differences among the sub-sub-sub, sub-basic-sub, sub-sup-sub. *p* > 0.05 [one-way repeated-measures analysis of variance (ANOVA)]. **(B)** Accuracies in response to target stimuli in the four conditions. There were no significant differences among the sub-sub-sub, sub-basic-sub, sub-sup-sub. *p* > 0.05 [one-way repeated-measures analysis of variance (ANOVA)], *N* = 18. For each condition, error bars represent ±SEM across participants. NB. “RT” is response time; “sub-sub-sub,” “sub-bas-sub,” and “sub-sup-sub” represent subordinate-subordinate-subordinate, subordinate-basic-subordinate, and subordinate-superordinate-subordinate, respectively.

Figure 2 shows the descriptive statistics of RTs and accuracy for the three experimental (sub-sub-sub, sub-bas-sub, sub-sup-sub) conditions. We conducted three-level one-way repeated-measures ANOVAs on mean RTs and accuracy. For RTs, there were no significant differences among the three levels (*p* > 0.05); therefore, RTs were not analyzed further. A significant difference was found on accuracy among the three conditions (*F*_(2,17)_ = 4.57, *p* < 0.05, *η*^2^ = 0.21. *Post hoc* tests revealed no significant differences between any two conditions (*p* > 0.05).

### ERP Results

**Figure 3:**
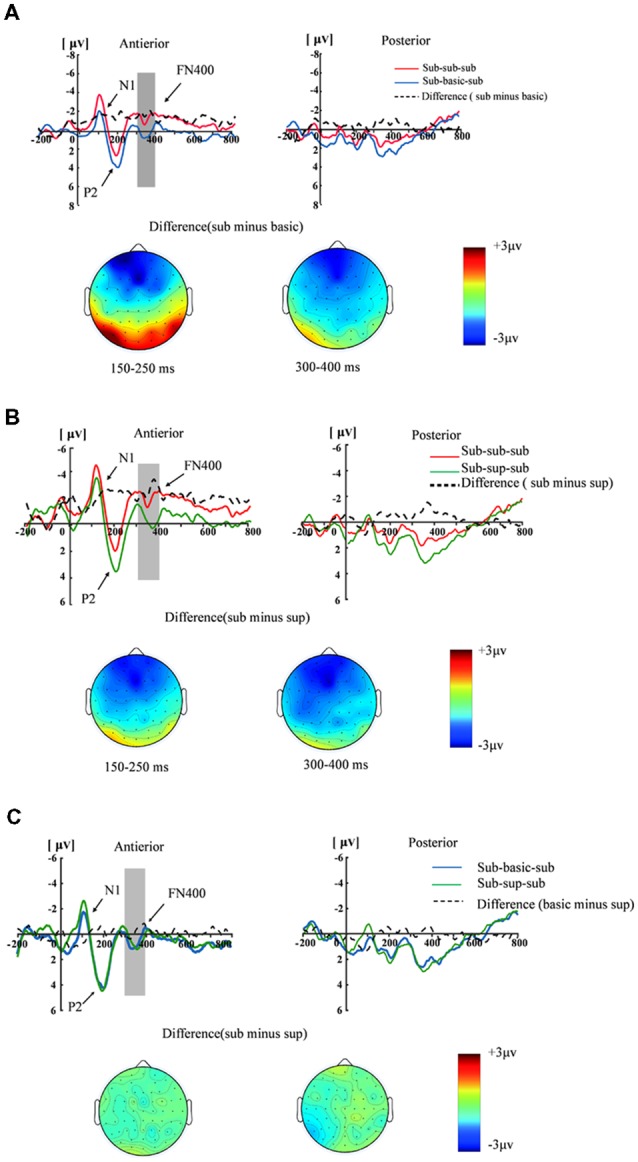
Grand-average event-related potential (ERP) waveforms measured at the anterior [(F3+F1+Fz+F2+F4+FC3+FC1+FCz+FC2+FC4)/10] and posterior [(CP3+CP1+CPZ+CP2+CP4+P3+P1+Pz+P2+P4)/10] regions for the subordinate masking, basic masking and subordinate-superordinate-subordinate. **(A)** The grand-averaged waveforms elicited by subordinate masking stimulus and basic masking stimulus and the difference waveforms (subordinate minus basic) in the anterior and posterior regions, as well as the topographies of the difference waveforms at 150–250 ms and 300–400 ms. **(B)** Grand-averaged waveforms elicited by subordinate masking stimulus and superordinate masking stimulus and the difference waveforms (subordinate minus superordinate) in the anterior and posterior regions, as well as the topographies of the difference waveforms at 150–250 ms and 300–400 ms. **(C)** Grand-averaged waveforms elicited by basic masking stimulus and superordinate masking stimulus and the different waveforms at 150–250 ms and 300–400 ms.

Figure 3 illustrates the ERP component for three conditions. As for N1 amplitudes (50–150 ms), a significant two-way interaction of masking type × brain region (*F*_(2,17)_ = 5.22, *p* < 0.05, *η*^2^ = 0.24). A significant region effect was found (*F*_(1,17)_ = 5.17, *p* < 0.05, *η*^2^ = 0.23). The anteriority analysis revealed a significant masking type effect: the N1 was larger for subordinate-subordinate-subordinate condition than by subordinate-basic-subordinate condition (*F*_(2,17)_ = 4.50, *p* < 0.05, *η*^2^ = 0.21). As for P2 amplitudes (150–250 ms), the interaction between masking type and brain region was significant (*F*_(2,17)_ = 7.27, *p* < 0.01, *η*^2^ = 0.30). A main effect of masking type was found in anterior region (*F*_(2,17)_ = 12.81, *p* < 0.001, *η*^2^ = 0.43). The subordinate-basic-subordinate condition and the subordinate-superordinate-subordinate condition elicited larger P2 amplitudes than the subordinate-subordinate-subordinate condition. There was no significant difference between the subordinate-superordinate-subordinate condition and the subordinate-basic-superordinate condition (*p* > 0.05). For FN400 amplitude (300–400 ms), the interaction between masking stimuli type and brain region was significant (*F*_(2,17)_ = 4.15, *p* < 0.05, *η*^2^ = 0.19). A main effect of masking type was found in anterior region (*F*_(2,17)_ = 6.38, *p* < 0.01, *η*^2^ = 0.273). The subordinate-basic-subordinate condition and the subordinate-superordinate-subordinate condition elicited smaller N400 amplitudes than the subordinate-subordinate-subordinate condition. No significant difference on FN400 amplitude between the subordinate-superordinate-subordinate condition and subordinate-basic-subordinate condition (*p* = 0.59) was observed.

## Discussion

The present study yields new insights into how the relationship between mask and target may affect priming, fluency and familiarity. As far as we know, our study is the first to show that priming and familiarity quality can be reduced when target is highly similar to mask. We used a masked repetition priming paradigm and three types of masks: subordinate-level, basic-level, and superordinate-level. Our behavioral results showed no effect of mask type on RT and accuracy. However, our ERP results showed that the conceptual hierarchical related masked repetition priming was associated with early anterior N1, P2 and FN400.

Although we previously predicted a different behavioral response (e.g., a reduction of recognition accuracy and the increase in RT in highly similar pairs) among three conditions, our behavioral results did not show any effect on the priming and familiarity. However, the ERP results indicated that the relationship between mask and target has successfully reduced the fluency and further attenuated the priming and familiarity. Li et al. (2015) interpreted this inconsistency between behavioral and ERP data as the behavioral response is less sensitive than ERP response in detecting the effect of processing fluency on subsequent familiarity. As this interpretation was not directly confirmed by them, and the different tasks that were used in our study, further evidences are needed to investigate whether the hierarchical relationship between the mask and target can indeed affect fluency and subsequent priming and familiarity in the behavioral dimension.

The current ERP time-course analysis showed that different conceptual feature between mask and target modulate processing of the target at N1 time window (50–150 ms): larger N1 for target following subordinate level mask than the basic level mask. The consistency with previous studies (Hoenig et al., 2008; Lin and Chan, 2018) is that the N1 component is sensitive measuring the within category feature difference instead of between category feature (for review please see the “Introduction” section). Inconsistently, previous study directly presented the characteristic attributes of words as the priming stimulus to see the variation of the target (e.g., round-orange, Hoenig et al., 2008; e.g., for self-defense-knife, Lin and Chan, 2018). Nevertheless, our study used the concept hierarchical related word as the mask to manipulate the affection to target (e.g., fruit-apple). In this case, the features are needed to be extracted first before further processing. Our results here may indicate that N1 is not only related to feature categorization but also feature extraction. The more shared features, the more cognitive resources were needed to distinguish the mask and target, which led to a largest N1 in the sub-sub-sub condition.

It was also noted that the subordinate level mask showed a smallest N1 effect, while the superordinate level did not differ from either subordinate or basic level. The reduction in processing basic level might be due to the basic level superiority effect which refer to the phenomenon that basic level is cognitively optimal for perception, categorization, communication, and knowledge organization and episodic information (Rosch et al., 1976; Large et al., 2004; Pansky and Koriat, 2004). In simpler terms, it means we tend to process objects’ information in basic level. For example, the basic-level category is typically the answer when we name an object (Jolicoeur et al., 1984). Hence, the basic level requires less cognitive resource to distinguish the concept feature than compared to other level, which could result in a smaller N1 amplitudes.

The main goal of the current experiment was to investigate the interaction of the putative electrophysiological markers of how hierarchical relationship between mask and target affect conceptual fluency, priming and familiarity. According to the P2 results, trials with low similar mask-target pairs (i.e., the sub-basic-sub and the sub-sup-sub trials) evoked larger P2 amplitudes than did those with high similar mask-target pairs (i.e., the sub-sub-sub trials). This early effect persisted with similar topography into 300–400 ms time window. Specifically, the sub-sub-sub condition produced larger negative FN400 component than sub-bas-sub and sub-sup-sub condition. The results are consistent with the assumption that the highly similar word pairs attenuated the priming and the familiarity effects. There are two explanation for the form of enhanced P2 and attenuated FN400 for target words. From the priming aspect, this effect was only observed for immediate masked repetition priming (Misra and Holcomb, 2003), and this is consist with current study. The author explained this as the automatic and implicit processing of the prime. But this interpretation fuzzed the specific role of P2 in the priming effect. According to the interpretation, our results might reflect that the similarity between the mask and target can affect priming effect, and highly similar pairs can impair this effect. From the familiarity aspect, Bader and Mecklinger (2017) found that new words were significantly more larger than old words. Combined with previous repetition priming study (Voss and Paller, 2009; Li et al., 2015), they suggested that the P2 reflect the perceptual fluency, which may source from the oldness and priming. In contrast, in the current study, the similarity of mask and target may reduce the perceptual fluency, which lead a small priming and familiarity effect.

In the current study, as can be seen from Figure 3, the topographical distribution of mid-frontal old/new effect did not differ from that of the priming N400 (Stróżak et al., 2016). Previous study used old/new judgment to investigate the familiarity, in addition they found the “old” response was facilitated when the test cues were primed by the same word when the old judgments were associated with “know” response (e.g., Rajaram, 1993; Woollams et al., 2008). In the present study, we conducted a semantic congruency judgment, which was a well-established categorization method to investigate the unconsciously word priming effect (Dehaene et al., 1998; Ortells et al., 2016). Although categorization tasks were also related to familiarity effect (Bruett and Leynes, 2015; Leynes et al., 2017), future study should adopt a typical familiarity task such as old/new judgment, or knew/remember judgment to insurance the purpose. Given that the semantic priming task and the recognition task can only partially distinguish the priming and familiarity effect (Stróżak et al., 2016), further evidence is required to decompose these functionally distinct processes. This may be done by elucidating how the relationship between prime and mask and the relationship between mask and target may affect the FN400 effect.

Notably, although the sub-sub-sub condition elicits larger P2 and smaller FN400 than the sub-bas-sub and the sub-sup-sub conditions, no significant difference was found between the latter two conditions. Our results were somewhat similar to those of a recent masked priming study (Ortells et al., 2016). In that study, the authors tested how the relationship between prime and target (unrelated vs. weakly related vs. strongly related) affected behavioral (RT) and EEG (N400) responses. They found that the strongly related prime and target trigger significant larger priming effect than do the weakly related and the unrelated prime and target; however, no significant difference is found between the latter two conditions (similar behavior results also see Van den Bussche et al., 2012; Ortells et al., 2013). In addition, in an earlier word recognition study, Holcomb and Grainger (2006) manipulated words’ repeatability by having the target word fully or partially repeat the prime or is completely unrelated to the prime. They found that the partial repetition condition did not differ from the no-relation condition in N400 amplitude. All these findings suggest that unconscious priming and recognition may not be sensitive enough to differentiate the effect of low similar prime with that of no-relation prime. The reason might be that, in order to make quick and accurate responses (i.e., congruent or incongruent judgment), people may easily fail to distinguish “not very congruent” stimuli from incongruent stimuli, without being told to distinguish stimuli as meticulous as possible.

Our study is different from previous studies in several ways. First, we mainly discuss the hindrance of similarity between mask and target while previous studies aim to explore the promotion effect of similarity between prime and target. Second, we fail to observe any significant behavioral results while previous studies have observed significant behavioral results (Ortells et al., 2013, 2016). There should be a longer RT and a lower accuracy for the sub-sub-sub condition than for the sub-bas-sub and the sub-sup-sub conditions. Future research on this topic should examine whether there is effect on behavioral indicators.

## Conclusion

In summary, we used the masked repetition paradigm and we manipulated the similarity between mask and prime to investigate how mask-prime similarity can affect word priming and recognition. First, larger N1 was found in sub-sub-sub condition, it may reflect that more cognitive resources are needed to distinguish similar mask and target pairs. Second, we found the similarity effect in P2 and FN400: trials with high mask-prime similarity (i.e., sub-sub-sub) induces larger P2 (150–250 ms) and smaller mid-frontal FN400 amplitudes than do trials with low mask-prime similarity (i.e., sub-basic-sub and sub-sup-sub). Our results suggest that the hierarchical relation between mask and prime can recede fluency, priming and familiarity.

## Author Contributions

YL conceived and designed the experiments, contributed reagents, materials, and analysis tools. YL and YD performed the experiments. YL and YM analyzed the data. YL, YM and YD wrote the article.

## Conflict of Interest Statement

The authors declare that the research was conducted in the absence of any commercial or financial relationships that could be construed as a potential conflict of interest.

## Footnotes

^1^www.cncorpus.org
